# Mr. Howard, on Digitalis in Dropsy

**Published:** 1801-11

**Authors:** J. S. Howard

**Affiliations:** Member of the Royal College of Surgeons, London. Hull


### *To the Editors of the Medical and Physical Journal.*

GENTLEMEN,

THE following cafes of Dropfy, cured by Digitalis Purpurea, fhould they meet your approbation, I requeft you to infert in your valuable publication.

I am, &c.

J. S. HOWARD,
Member of the Royal College of Surgeons, London.

*Hull, Oct.* 1, 1801.

---

CASE. I.—On the 8th of April I was requefted to fee Mary A—n, whom I found labouring under afcites and anafarca. She informed me, fhe had been afflicted with a flight dry cough for many years; at the commencement of March fhe was feized with a diarrhœa, the prevailing epidemic, which continued for a fortnight, at the fame time fhe complained of very very great pain in the hypogaſtric region, which ſhe compared to that of child birth; when the diarrhœa ceaſed, her abdomen began to ſwell from the inferior part; at this time ſhe voided but a ſmall quantity of urine, which was of a red colour. In the right hypochondriac region was a large hard tumour, which extended down as low as the umbilicus; there was a very evident fluctuation in the cavity of the abdomen, with great oppreſſion in breathing: She was not able to lay upon her left ſide; her legs were alſo anaſarcous; at firſt I ordered her the the following formula:

℞. Calomel pta. gr. x. Pulv. radic. ſcillæ ʒj. Sapon Venet. ʒj. Syr. zinzib. q. s. fiat maſſa & divid in pilulas xvj. Capiat tres ter de die.

On the 10th her ſymptoms were nearly the ſame; her pulſe more frequent and hard.

12th. She was ſeized with a diarrhœa, and griping pains in the inteſtines; as the calomel was ſuppoſed to have produced this effect, a mercurial bolus with opium was ſubſtituted, *hora somni.*

13th. The pain in the inteſtines and diarrhœa were abated.

14th. She appeared much relieved.

15th. She was much worſe; breathing very urgent, with a ſtrong pulſe. Ordered her the following:

℞. Fol. digitalis purpurea ʒij. Infunde ex aq. bullient ℔ſs. ad horas duas cola adde ſpir. æther. nitros ℥j. Ft. mixtura ſum. coch. iij. 4tis horis.

16th. Breathing leſs troubleſome; had voided a large quantity of urine.

17th. Much as yeſterday. Rep. mixtura.

18th. Much better. Rep. mixt.

19th. The tumour of the abdomen entirely gone.

20th. Continues recovering; voids large quantities of urine, but rather coſtive. Ordered an aperient draught, with infus. ſennæ.

21ſt. Diſcontinued the mixture; ordered a nouriſhing diet, and a decoct. cortic flav. which ſhe continued taking till the 3d of May; her body reduced to its natural ſize, and every other ſymptom of aſcites and anaſarca gone; ſtill perſevered in the uſe of the cinchona till the 12th inſtant, when ſhe found herſelf quite recovered.

Case II.—Thomas R—n, aged ten years, I attended on the firſt of May; found his legs extremely œdematous, with a fluctuating hard abdomen; tenſe pulſe, continual thirſt, and voiding very little urine, with every other ſymptom of anaſarca. Gave him the following:

℞. Tinct.

℞. Tinct. digitalis purpurea ʒiij. Aqua cinnamomi ℥iv. Spir. nitri dulcis ℥ſs. M. cap. coch. j. 3tis horis.

Second day. Complains of violent pain in the inteſtines; ordered pulv. opiat. (P. N.) gr. xv. ſtatim capiendus, and to continue the mixture.

3d. Much as yeſterday. Cont. mixt.

4th. Bowels eaſy; voided near a quart of urine during the laſt twelve hours. Cont. mixt. et p. anod.

6th. Much better; legs nearly their natural ſize.

7th. Continues better; voids a large quantity of urine.

9th. Still better; omitted the mixt. and ſubſtituted decoct. cinch. flav. which was continued for ten days, when he was quite recovered.

